# Plasma Lysophosphatidylcholine and Lysophosphatidylethanolamine Levels Were Associated With the Therapeutic Response to Olanzapine in Female Antipsychotics-naïve First-episode Patients With Schizophrenia

**DOI:** 10.3389/fphar.2021.735196

**Published:** 2021-09-16

**Authors:** Jiahong Liu, Meihong Xiu, Haixia Liu, Jun Wang, Xirong Li

**Affiliations:** ^1^ The Affiliated Kangning Hospital of Wenzhou Medical University, Wenzhou, China; ^2^ Peking University HuiLongGuan Clinical Medical School, Beijing HuiLongGuan Hospital, Beijing, China; ^3^ Department of Psychiatry, Shandong Mental Health Center, Jinan, China

**Keywords:** schizophrenia, lysophospolipid, therapeutic response, olanzapine, association

## Abstract

**Background:** Accumulating studies have shown that the pathophysiology of schizophrenia may be associated with aberrant lysophospolipid metabolism in the early stage of brain development. Recent evidence demonstrates that antipsychotic medication can regulate the phospholipase activity. However, it remains unclear whether lysophospolipid is associated with the therapeutic response to antipsychotic medication in schizophrenia. This study aimed to investigate the influence of olanzapine monotherapy on lysophosphatidylcholine (LPC) and lysophosphatidylethanolamine (LPE) and the association between symptom improvement and changes of LPC and LPE levels during treatment in antipsychotic-naïve first-episode (ANFE) patients.

**Materials and Methods:** The psychotic symptoms were evaluated by the Positive and Negative Syndrome Scale (PANSS). 25 ANFE patients were treated with olanzapine for 1 mo. The levels of LPC and LPE were determined and psychotic symptoms were assessed at baseline and at 1-mo follow-up.

**Results:** Relative to baseline, the psychotic symptoms were significantly reduced after olanzapine treatment, except for negative symptoms. Moreover, the levels of most LPC and LPE increased after treatment. Interestingly, increased LPC(18:3) and LPC(20:2) levels were positively associated with the reduction rates of PANSS positive subscore. In addition, baseline levels of LPE(20:5), LPE(18:3) and LPE(22:5) were predictors for the reduction of positive symptoms.

**Conclusion:** Our study reveals that the levels of lysophospolipid are associated with the improvement of positive symptoms, indicating that LPC may be a potential therapeutic target for olanzapine in schizophrenia. Moreover, baseline LPE levels were predictive biomarkers for the therapeutic response to olanzapine in the early stage of treatment in ANFE patients.

## Introduction

Schizophrenia is a chronic and severe mental disorder affecting approximately 1% of the population (Barnett 2018). Currently, the first-line therapy for schizophrenia is atypical antipsychotic medication, such as olanzapine, risperidone and ziprasidone (Lieberman 1996). However, the therapeutic response to antipsychotics is heterogeneous between individuals. Current symptom-driven treatment leads to the poor outcome in patients with schizophrenia, especially in the early stage of treatment (Liu-Seifert et al., 2005).

Metabolites are the final products of the biochemical pathways in the human body, and their abnormalities can better reflect the disruption of functional status of the patients (Lindon et al., 2003). Yet, most of small-molecule metabolites are currently difficult to separate and detect (Rojo et al., 2012). Therefore, their roles in the pathophysiology of schizophrenia remain unclear. Recently, liquid chromatography tandem mass spectrometry (LC-MS) based metabolomics provided an opportunity to understand the pathological role of metabolites and develop new predictive biomarkers that can monitor the response to antipsychotics (Kaddurah-Daouk et al., 2008; Paredes et al., 2014; Pickard 2015). Our previous study by metabolomics method showed that olanzapine treatment for 1 month significantly increased the plasma levels of several types of lysophosphatidylcholine (LPC) and lysophosphatidylethanolamine (LPE) (Liu et al., 2021).

LPC and LPE are the prominent parts of lysophospolipid and play key roles in physiological and pathological processes of nervous system (Steinbrecher et al., 1984). Increasing evidence shows that LPC and LPE are involved in the function of cell membrane, apoptosis, oxidative stress and inflammatory responses. In the body, the peripheral circulating LPE and LPC are produced from hepatic secretion following the hydrolysis of cellular membrane phosphatidylcholine (PC) and phosphatidylethanolamine (PE) catalyzed by both acyltransferases and phospholipase (PLA) (Schmitz and Ruebsaamen 2010). In contrast, the majority of plasma LPC and LPE species may originate from liver secreted lecithincholesterol acyltransferases (LCAT) reaction (Sekas et al., 1985). In mechanism, LPC and LPE serve as a reservoir for arachidonic acid and as a central precursor for lipid-associated signaling molecular. In addition, LPC and LPE are bioactive lipids involved in monocyte recruitment and activation (Park et al., 2015). For example, studies found that LPC and LPE have protective role on ischemic neurons during the oxidative stress (Blondeau et al., 2002; Chen et al., 2019).

Schizophrenia shows complex and unique abnormal characteristics of glycerophospholipids in serum, plasma or brain tissues (Horrobin et al., 1994; Schmitt et al., 2004; He et al., 2012; Ghosh et al., 2017). Non-targeted LC-MS based metabolic profiling studies in schizophrenia spectrum disorders suggest altered glycerophospholipid species and levels in the brain of patients (Fukuzako et al., 1999; Matsumoto et al., 2011). Metabolomics study of prefrontal cortex from schizophrenia patients reported significantly lower levels of PCs in white and grey matter than healthy controls (Schwarz et al., 2008). Further, some studies revealed that after antipsychotics treatment, schizophrenia patients showed increased levels of 22:5, 20:5, and 20:3 within the PC and PE glycerophospholipid classes (Kaddurah-Daouk et al., 2012; Castillo et al., 2016). Particularly, a study comparing the lipid profiles of antipsychotics-free and drug-naïve (ANFE) patients before and after several types of antipsychotics medication for 7 mo showed that 11 glycerophospholipids (nine PCs and two LPCs) were significantly up-regulated after treatment, suggesting that glycerophospholipids may be used for predictive markers to monitor the treatment in the early stage of this disorder (Leppik et al., 2020).

Based on the aforementioned literatures regarding the phospholipids in schizophrenia, we hypothesized that the LPC and LPE levels were associated with the improvement of clinical symptoms after 1-month treatment. Therefore, this study aimed to investigate whether the plasma LPC and LPE levels identified by LC-MS based metabolomics were the predictive markers for the treatment response to olanzapine in the early treatment of ANFE patients with schizophrenia.

## Methods

### Subjects

Female ANFE patients with schizophrenia diagnosed as DSM-IV criteria and confirmed by the Structured Clinical Interview for DSM-IV (SCID) were recruited. The inclusion criteria were described in our previous study. In brief, *1*) female Han Chinese; *2*) between 18 and 45 yr old; *3*) antipsychotic free or cumulative antipsychotic treatment <14 days; *4*) without substance abuse, including alcohol and smoking. We acquired a complete physical examination and medical history from all patients to exclude serious physical conditions. A detailed questionnaire including general information, sociodemographic characteristics, and medical and psychological conditions was administered to each subject by a member of the research staff. Additional information was collected from available medical records. The average age and onset age of the ANFE patients were 27.4 ± 7.6 and 26.4 ± 8.9. The mean year of education was 9.1 ± 3.5 and mean BMI was 21.0 ± 3.0 (shown in Table 1).

**TABLE 1:** Reduction of psychotic symptoms assessed by PANSS after treatment with olanzapine for 4 wk.

	Baseline	Follow-up	Reduction rate	*p* value^a^
Age (years)	27.4 ± 7.6			
Education (years)	9.1 ± 3.5			
BMI (kg/m^2^)	21.0 ± 3.6			
Onset age (years)	26.4 ± 8.9			
Clinical symptoms				
P subscore	25.0 ± 6.0	16.4 ± 5.2	0.33	<0.001
N subscore	17.1 ± 4.7	15.6 ± 4.3	0.04	0.23
G subscore	39.7 ± 7.5	32.0 ± 6.0	0.17	<0.001
Total score	81.8 ± 13.9	63.9 ± 13.8	0.21	<0.001

*PANSS* the positive and negative syndrome scale.

a Comparison of psychotic symptoms between baseline and follow-up.

The study was approved by the Institutional Review Board of Beijing Huilongguan Hospital, and all patients provided the written informed consent.

### Study Procedures

It is an open-labeled, single center, longitudinal observational study. ANFE patients were treated with a flexible-dosed (ranged from 10 to 30 mg/day) olanzapine for 1 mo. All patients stayed in hospital and the nurses monitored the adherence to olanzapine treatment throughout the study.

### Clinical Symptom Assessment

The Positive and Negative Syndrome Scale (PANSS) was used to evaluate the severity of clinical symptoms by three experienced psychiatrists. Each patient was assessed on PANSS scale at baseline and at 1-mo follow-up. The equation used to calculate the rate of the clinical symptom reduction was (T_b_-T_a_)/T_a_×100%, where T_a_ is the score at baseline and T_b_ is the score at follow-up.

### Plasma Processing and Metabolomics Data Processing

Venous blood samples from participants were collected at two time points after fasting: on admission and 1-mo follow-up. Plasma samples were separated after centrifugation for 15 min at 4°C and stored at −80°C until further processing. The sampling process was described in our previous study (Liu et al., 2021). In brief, 200 μl of plasma was added to a vial, extracted with methanol (600 μl), and centrifuged for 2 min. The supernatant was absorbed and dried in nitrogen at 37°C. Then, the treated supernatant was analyzed using a UPLC-MS metabolomics (Liang et al., 2021; Liu et al., 2021). MS analysis was carried out in positive ion mode. We obtained scans in the mass range of 70–1000 m/z, at three scans per second with a resolution of 70,000. For the MS/MS assay, a normalized collision energy of 35 V, an isolation window of 0.8 m/z, and a mass resolution of 35,000 were used.

Data acquisition from the raw MS output files were achieved using XCALIBUR software (Thermo Fisher Scientific). Progenesis QI (Waters) was conducted to extract the mass spectral features. Principal component analysis (PCA) was conducted using SIMCA-P 13 software (Umetrics). Partial least squares discriminate analysis (PLS-DA) model was performed for the calculation of variable importance in projection (VIP) values. Annotated compounds were identified by searching the accurate mass of the molecular ions and the fragment ions against compound databases.

### Statistical Analysis

All the analyses were performed by using SPSS 20.0. The Kolmogorov-Smirnov (K-S) test was performed to test the normality of the relative intensity of the metabolites. Comparisons of plasma metabolites between baseline and follow-up were conducted with paired *t*-test. The analysis of variance (ANOVA) was performed for the group comparison of continuous variables. In addition, Pearson product moment correlation was conducted to analyze the correlations between the rate of reduction of PANSS and the increase in the relative intensity of LPC and LPE. Multiple linear regression analysis was performed to adjust for the confounding factors. In this model, the reduction percentage of PANSS score was the dependent variable and the demographic data and the increase of LPC and LPE were the independent variables. Bonferroni corrections were applied for multiple testing. The significance levels were set at *p* < 0.05.

## Results

### Psychotic Symptoms at Baseline and After Treatment

Initially, we tested 27 ANFE patients using LC-MS metabolomics approach. Only 25 patients had complete LPC and LPE data, which were included in the following analysis. Table 1 shows the psychotic symptoms of 25 ANFE patients at baseline and 1-mo follow-up. After 1 mo of olanzapine monotherapy, patients showed a significant improvement in the psychotic symptoms (all *p*_Bonferroni_<0.05), except for negative symptoms. The mean reduction rate of psychotic symptoms ranged from 0.04 (negative subscore) to 0.33 (positive subscore).

### LPC and LPE Levels at Baseline and 1-mo Follow-up

**FIGURE 1:**
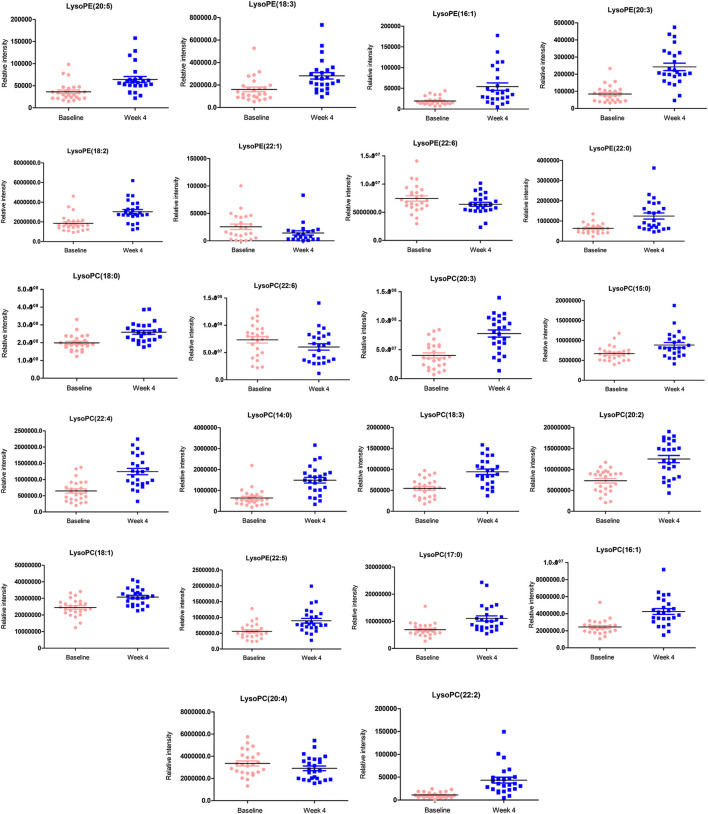
This figure presents the boxplots of the metabolite levels at baseline and at follow-up after treatment with olanzapine for 1 mo. The sample means are indicated by the black bars. The levels of most lysophosphatidylcholine (LPC) and lysophosphatidylethanolamine (LPE) were significantly increased after treatment with olanzapine (all *p* < 0.01).

We identified 13 LPC and nine LPE in the differential compound library. The relative intensities of these metabolites were all normally distributed (K-S test: all *p* > 0.05). As shown in Table 2 and Figure 1, the levels of most LPC and LPE were significantly increased after treatment with olanzapine (*p* < 0.01). However, LPE(22:1) and LPC(22:6) levels were significantly decreased (all *p* < 0.05), and LPE(22:6) and LPC(20:4) showed no significant difference after treatment (all *p* > 0.05).

**TABLE 2:** Changes of metabolites from baseline to 4-wk follow-up (Paired *t* test).

Metabolites	Regulation	Changes from baseline (95% CI)	*p*	Fold changes
LysoPE(20:5)	Up	28008 (10889, 45128)	0.002	0.92 (0.17, 1.35)
LysoPE(18:3)	Up	120565 (36447, 204683)	0.007	0.90 (0.01, 2.35)
LysoPE(16:1)	Up	34625 (15665, 63639)	0.001	1.001 (0.16, 3.09)
LysoPE(20:3)	Up	158801 (110956, 206645)	<0.001	1.36 (0.57,3.87)
LysoPE(22:5)	Up	332257 (199231, 465282)	<0.001	0.54 (0.17, 1.22)
LysoPE(18:2)	Up	1180553 (595466, 1765639)	<0.001	0.72 (0.22, 1.41)
LysoPE(22:1)	Down	−12508 (−18344, −6672)	<0.001	−0.5 (−0.86, −0.22)
LysoPE(22:6)	No change	−1022620 (−2116118, 70878)	0.07	−0.14 (−0.28, 0.10)
LysoPE(22:0)	Up	613281 (300488, 926074)	<0.001	0.55 (0.17, 1.56)
LysoPC(18:0)	Up	59496023 (28783987, 90208060)	0.001	0.17 (−0.08, 0.39)
LysoPC(22:6)	Down	-13073032 (-24702772, -1443292)	0.03	−0.15 (−0.45, 0.17)
LysoPC(20:3)	Up	37830985 (28049234, 47612735)	<0.001	0.98 (0.53, 1.54)
LysoPC(20:4)	No change	−437356 (−908067, 33353)	0.07	−0.14 (−0.30, 0.09)
LysoPC(15:0)	Up	2149849 (729001, 3570697)	0.005	0.30 (0.01, 0.46)
LysoPC(22:4)	Up	597175 (380502, 813847)	<0.001	1.26 (0.34, 1.88)
LysoPC(17:0)	Up	4130458 (1999412, 6261504)	0.001	0.43 (0.08, 10.9)
LysoPC(14:0)	Up	850540 (614758, 1086323)	<0.001	1.16 (0.65, 2.29)
LysoPC(16:1)	Up	1819062 (1271635, 2366489)	<0.001	0.81 (0.33, 1.23)
LysoPC(18:3)	Up	395471 (232238, 558705)	<0.001	0.63 (0.25, 1.40)
LysoPC(20:2)	Up	5180523 (3697696, 6663350)	<0.001	0.59 (0.39, 1.02)
LysoPC(22:2)	Up	32447 (19464, 45431)	<0.001	2.49 (1.45, 4.65)
LysoPC(18:1)	Up	6273223 (3923344, 8623102)	<0.001	0.25 (0.09, 0.45)

PC, phosphatidylcholine; PE, phosphatidylethanolamine; CI, confidence. Change refers to the levels of metabolites at follow-up minus the levels at baseline.

### Associations Between Changes in Metabolites and Improvement of Symptoms From Baseline

**FIGURE 2:**
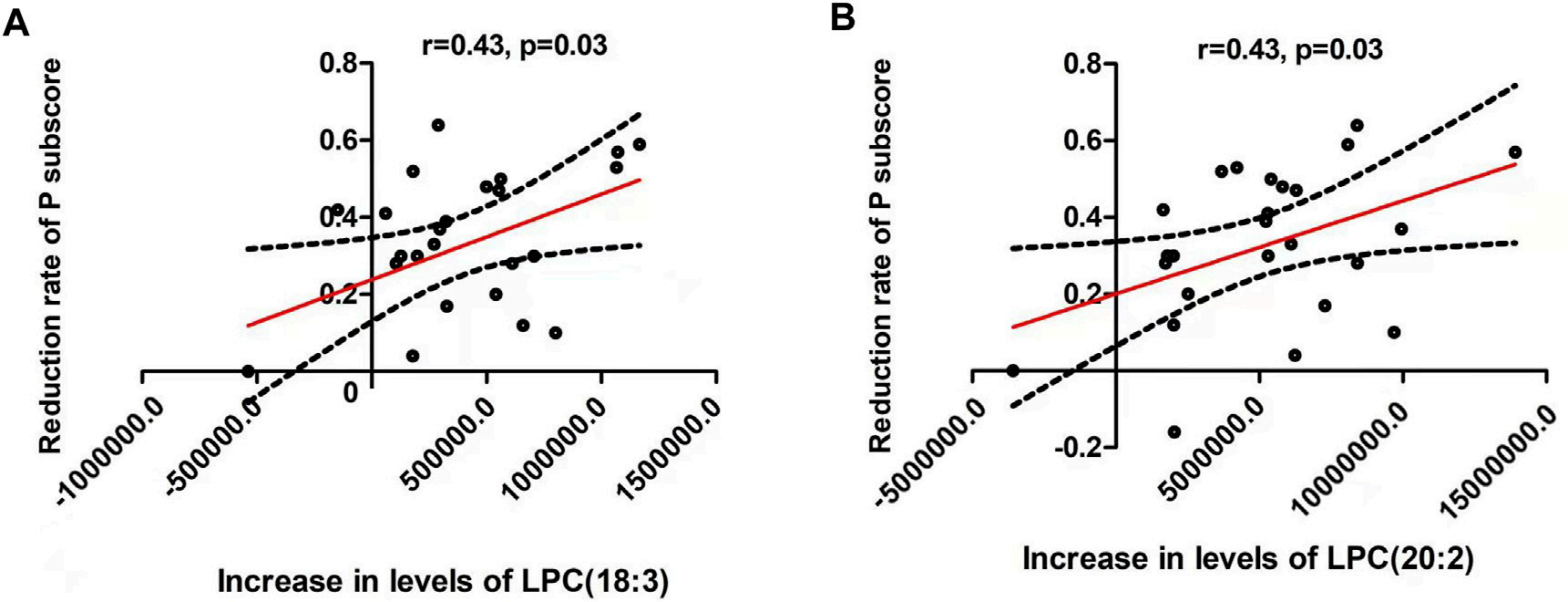
There were significant positive associations between the reduction of positive symptoms and the increase of lysophosphatidylcholine (LPC) (18:3) **(A)** and LPC(20:2) **(B)** (both were: *r* = 0.43, *p* = 0.03). The red line represents the trend line, and the dashed black line is the 95% confidence interval.

The increases of LPC(18:3) and LPC(20:2) were positively associated with the reduction rates of PANSS positive subscore (both were: *r* = 0.43, *p* = 0.03) (Table 3 and Figure 2). Moreover, the increase of LPC(18:3) was significantly associated with the increase of LPC(20:2) (*r* = 0.65, *p* < 0.001). Further regression analyses confirmed the relationship between the reduction rate of PANSS positive subscore and the increase of LPC(18:3) (β= 0.68, t = 3.1, *p* = 0.006; *R* ^2^ = 0.41) or LPC(20:2) (β= 0.56, t = 2.5, *p* = 0.02; *R* ^2^ = 0.33) after controlling for age, education, baseline BMI, onset age and the increase of BMI.

**TABLE 3:** The correlation between the increase of metabolite levels and the reduction of clinical symptoms.

Increase of metabolites	P subscore (r/p)	N subscore (r/p)	G subscore (r/p)	Total score (r/p)
LysoPE (20:5)	0.17 (0.41)	−0.14 (0.50)	0.17 (0.41)	0.12 (0.56)
LysoPE (18:3)	0.25 (0.23)	−0.11 (0.61)	0.12 (0.56)	0.12 (0.58)
LysoPE (16:1)	−0.18 (0.39)	0.06 (0.79)	0.09 (0.67)	−0.01 (0.97)
LysoPE (20:3)	0.07 (0.73)	−0.10 (0.63)	0.26 (0.21)	0.16 (0.43)
LysoPE (22:5)	0.03 (0.88)	−0.13 (0.55)	0.15 (0.46)	0.07 (0.73)
LysoPE (18:2)	0.32 (0.12)	−0.09 (0.66)	0.23 (0.26)	0.22 (0.29)
LysoPE (22:1)	0.14 (0.51)	−0.10 (0.62)	0.11 (0.59)	0.12 (0.56)
LysoPE (22:6)	−0.04 (0.85)	−0.37 (0.07)	−0.14 (0.51)	−0.18 (0.38)
LysoPE (22:0)	−0.05 (0.82)	−0.11 (0.59)	0.01 (095)	−0.03 (0.88)
LysoPC(18:0)	0.06 (0.79)	0.05 (0.83)	−0.03 (0.91)	0.04 (0.84)
LysoPC(22:6)	0.04 (0.87)	−0.11 (0.60)	0.06 (0.77)	0.04 (0.84)
LysoPC(20:3)	0.25 (0.22)	−0.02 (0.91)	0.003 (0.99)	0.08 (0.71)
LysoPC(20:4)	−0.14 (0.51)	−0.17 (0.43)	−0.15 (0.48)	−0.17 (0.41)
LysoPC(15:0)	−0.14 (0.50)	−0.23 (0.26)	−0.12 (0.56)	−0.20 (0.35)
LysoPC(22:4)	0.17 (0.41)	−0.10 (0.65)	0.02 (0.94)	0.05 (0.81)
LysoPC(17:0)	−0.23 (0.26)	−0.16 (0.45)	−0.02 (0.94)	−0.13 (0.52)
LysoPC(14:0)	−0.10 (0.63)	−0.09 (0.67)	0.09 (0.69)	−0.002 (0.99)
LysoPC(16:1)	−0.13 (0.53)	0.06 (0.78)	0.16 (0.46)	0.08 (0.72)
LysoPC(18:3)	0.43 (0.03)^a^	−0.04 (0.87)	0.18 (0.40)	0.26 (0.21)
LysoPC(20:2)	0.43 (0.03)^a^	−0.04 (0.86)	0.02 (0.94)	0.14 (0.51)
LysoPC(22:2)	−0.14 (0.50)	−0.13 (0.52)	−0.08 (0.70)	−0.14 (0.50)
LysoPC(18:1)	0.23 (0.26)	−0.33 (0.11)	−0.16 (0.46)	−0.10 (0.63)

a *p* < 0.05.

### Association Between Baseline Metabolites and the Improvement of Clinical Symptoms

**FIGURE 3:**
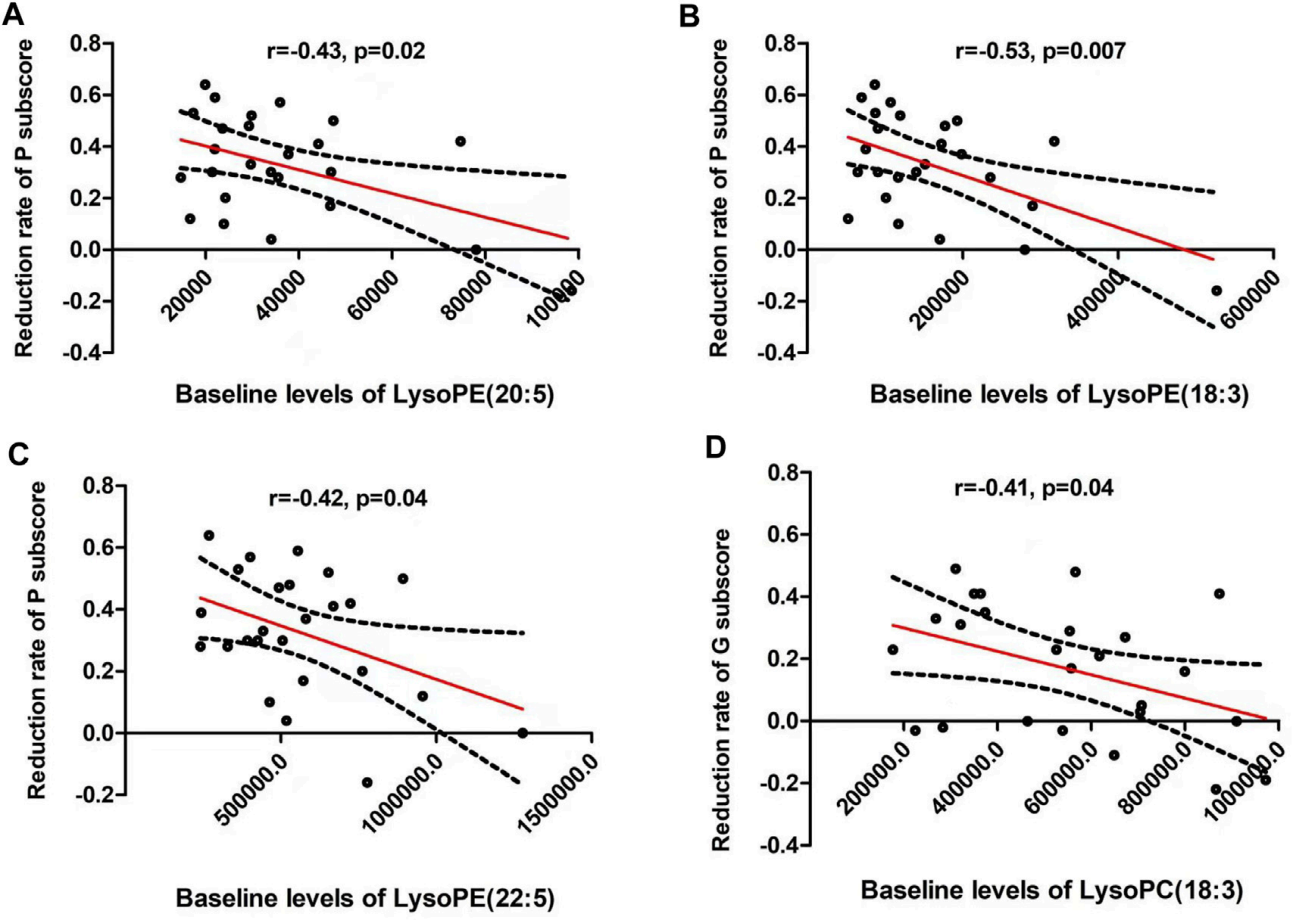
The reduction rate of PANSS positive subscore was negatively correlated with baseline lysophosphatidylethanolamine (LPE) (20:5) (*r* = −0.47, *p* = 0.02) **(A)**, LPE (18:3) (*r* = −0.53, *p* = 0.007) **(B)** and LPE (22:5) (*r* = −0.42, *p* = 0.04) **(C)**. In addition, LPC(18:3) was negatively correlated with the reduction rate of general psychological subscore (*r* = −0.41, *p* = 0.04) **(D)**. The red line represents the trend line, and the dashed black line is the 95% confidence interval.

The reduction rate of PANSS positive subscore was negatively correlated with baseline LPE(20:5) (*r* = −0.47, *p* = 0.02), LPE(18:3) (*r* = −0.53, *p* = 0.007) and LPE(22:5) (*r* = −0.42, *p* = 0.04) (Table 4 and Figure 3). Moreover, LPC(18:3) was negatively correlated with the reduction rate of general psychological subscore (*r* = −0.41, *p* = 0.04). Multiple regression analysis identified that baseline LPE(20:5) (β= −0.55, t = 2.7, *p* = 0.02; *R* ^2^ = 0.35), LPE(18:3) (β= −0.56, t = 2.9, *p* = 0.01; *R* ^2^ = 0.39) and LPE(22:5) (β= −0.58, t = 2.7, *p* = 0.01; *R* ^2^ = 0.36) were the contributing factors for the reduction rate of positive subscore.

**TABLE 4:** The correlation between the baseline metabolite levels and the reduction of clinical symptoms.

Baseline metabolite levels	P subscore (r/p)	N subscore (r/p)	G subscore (r/p)	Total score (r/p)
LysoPE (20:5)	**−0.47 (0.02)^a^**	0.11 (0.61)	−0.02 (0.93)	−0.12 (0.58)
LysoPE (18:3)	**−0.53 (0.007)^b^**	0.11 (0.61)	−0.05 (0.81)	−0.14 (0.50)
LysoPE (16:1)	−0.14 (0.49)	016 (0.43)	0.004 (0.99)	0.002 (0.99)
LysoPE (20:3)	−0.22 (0.30)	0.09 (0.66)	−0.09 (0.67)	−0.09 (0.66)
LysoPE (22:5)	**−0.42 (0.04)^a^**	0.12 (0.58)	−0.09 (0.68)	−0.16 (0.43)
LysoPE (18:2)	−0.57 (0.003)	0.08 (0.70)	−0.10 (0.65)	−0.21 (0.33)
LysoPE (22:1)	−0.04 (0.85)	0.09 (0.66)	−0.13 (0.53)	−0.09 (0.66)
LysoPE (22:6)	0.03 (0.90)	0.33 (0.11)	0.17 (0.42)	0.18 (0.38)
LysoPE (22:0)	0.17 (0.41)	0.19 (0.36)	0.14 (0.52)	0.20 (0.34)
LysoPC(18:0)	0.26 (0.20)	0.13 (0.54)	0.12 (0.56)	0.21 (0.32)
LysoPC(22:6)	0.01 (0.97)	0.07 (0.73)	−0.31 (0.13)	−0.20 (0.33)
LysoPC(20:3)	−0.13 (0.53)	−0.01 (0.98)	−0.33 (0.11)	−0.27 (0.19)
LysoPC(20:4)	0.07 (0.75)	0.03 (0.88)	−0.14 (0.49)	−0.09 (0.66)
LysoPC(15:0)	−0.02 (0.91)	0.28 (0.17)	−0.05 (0.81)	0.20 (0.93)
LysoPC(22:4)	−0.27 (0.19)	0.09 (0.68)	−0.38 (0.06)	−0.32 (0.12)
LysoPC(17:0)	0.20 (0.33)	0.24 (0.26)	0.09 (0.68)	0.19 (0.37)
LysoPC(14:0)	−0.05 (0.83)	0.09 (0.66)	−0.07 (0.75)	−0.02 (0.92)
LysoPC(16:1)	−0.06 (0.77)	0.17 (0.41)	−0.14 (0.51)	−0.07 (0.75)
LysoPC(18:3)	−0.38 (0.06)	0.02 (0.94)	−0.41 (0.04)*	−0.38 (0.06)
LysoPC(20:2)	0.02 (0.94)	0.12 (0.56)	−0.31 (0.13)	−0.18 (0.39)
LysoPC(22:2)	−0.07 (0.74)	0.14 (0.51)	−0.16 (0.45)	−0.09 (0.68)
LysoPC(18:1)	−0.29 (0.16)	0.27 (0.19)	−0.27 (0.20)	−0.22 (0.29)

a *p* < 0.05.

b *p* < 0.01.

## Discussion

This study found that schizophrenia patients showed increased concentrations of most of LPC and LPE after olanzapine monotherapy for 1 mo. Increased LPC(18:3) and LPC(20:2) levels were correlated with the improvement of positive symptoms. Baseline levels of LPE(20:5), LPE(18:3) and LPE(22:5) also were associated with the reduction rate of positive symptom and may serve as a predictive biomarker for therapeutic response.

Using LC-MS based untargeted metabolomics approach, abnormalities in levels of LPE and LPC have been consistently reported in schizophrenia, indicating that abnormal metabolism of LPE and LPC is a pathophysiological characteristic of this disorder (Law et al., 2019). Lysoglycerophospholipid plays a fundamental role in the neuronal mechanisms underlying the pathophysiological feature of several psychiatric disorders. Moreover, atypical antipsychotic medication has been shown to alter the levels of LPE and LPC in mental disorders (Ask et al., 2016; Cao et al., 2019; Leppik et al., 2020; Canfrán-Duque et al., 2021). In line with our findings, Cao et al. found that after 8-wk treatment with antipsychotics, serum amino acids and LPC levels were increased in first-episode (FEP) patients with schizophrenia (*n* = 122) (Cao et al., 2019). Leppik et al. profiled 14 LPCs and 76 PCs in serum from 53 FEP patients and found two LPCs and nine PCs levels were significantly increased after antipsychotic treatment for 7 mo (Leppik et al., 2020). Kaddurah-Daouk et al. found that PE levels were reduced in schizophrenia patients, and this change was reversed by olanzapine treatment (Kaddurah-Daouk et al., 2007). Particularly, Qiao et al. reported that decreased LPC (20:3) and LPC (14:0) levels were significantly increased after treatment with olanzapine for 1 mo in female patients with ANFE schizophrenia. However, only 13 metabolites were analyzed in this study (Qiao et al., 2016). The underlying mechanism of increase in the LPC and LPE levels after treatment with olanzapine in patients with schizophrenia is unclear. One possible explanation may be linked with the aberrant activity of phospholipase A_2_ (PLA_2_) after treatment. It is well known that PLA_2_ is a class of enzyme that catalyzes cleavage of fatty acids from the sn-2 position of phospholipids and hydrolyze phospholipids into LPE and LPC (Hishikawa et al., 2014). Several antipsychotics have been reported to influence the activities or levels of PLA_2_ in animal models and patients with schizophrenia (Gattaz et al., 1995; Smesny et al., 2005; Smesny et al., 2011; Kim et al., 2012; Shen et al., 2018). Our findings provide further evidence for the regulation of LPE and LPC concentrations by olanzapine treatment in patients with schizophrenia.

Our second finding is that the improvements of clinical symptoms after treatment were associated with the changes of LPC and LPE. The potential mechanism may be related to the physiologic functions of LPC and LPE, such as the formation of cell membranes, vesicle trafficking and bioactive signaling molecules in various cells including neuron (van Meer et al., 2008). As a component of lysophospholipid, LPC and LPE are water-soluble and amphiphilic molecules. They are essential precursors of signaling pathway molecules that activate second messenger to enhance the biological functions. Previous studies have shown that lysophospholipid can stabilize and enhance G protein-coupled receptor G2A signaling pathway for calcium flux by preventing its reuptake or altering its surface expression and localization on the cell surface (Wang et al., 2005; Frasch et al., 2007). There is robust evidence that inflammation and redox imbalance play a crucial role in the pathophysiology of schizophrenia (Zhang et al., 2017; Zhang et al., 2018; Xiu et al., 2019; Xiu et al., 2020). LPC and LPE have also been found to be associated with the regulation of redox system and inflammatory response systems. For example, Cui et al. found that plasma PC (36:4) was correlated with concentration of tumor necrosis factor alpha (TNF-α) and interleukin-6 (IL-6) in septic rat models (Cui et al., 2020). Carneiro et al. found that LPC triggered Toll-like receptors 2 (TLR2)- and TLR4-mediated signaling pathways but counteracted LPS-induced NO synthesis by inhibiting nuclear factor kappa-light-chain-enhancer of activated B (NF-κB) translocation and mitogen activated protein kinase/extracellular-signal-regulated kinase (MAPK/ERK) phosphorylation (Carneiro et al., 2013). In particular, a recent randomized, double-blind and placebo-controlled trial showed that a decomposition product of pig liver, which is a rich source of LPC and LPE, has been identified as a nutrient to improve cognitive function in the healthy individuals over the age of 40 (Matsuda et al., 2020). Moreover, administration with LPC and LPE on the lipopolysaccharides (LPS)- stimulated SIM-A9 microglia cells significantly reduced the expression of IL-6 and the production of reactive oxygen species (ROS) (Tsukahara et al., 2021). However, it remains unclear whether the increased levels of LPC and LPE are the causes or results of the improvement of clinical symptoms. The exact mechanism needs further study.

Specifically, we found that the different classes of lysophospholipids were involved in the improvement of clinical symptoms in this study. Baseline LPE levels including (20:5), (18:3) and (22:5) and the increase in LPC levels including (18:3) and (20:2) were associated with the clinical symptom improvements. In line with our findings, previous studies also supported that lysophospholipid with different group has different functional role. In the studies of prehypertension, increased LPC(16:0) was found to cause oxidative stress, thereby increases inflammation and arterial stiffness (Kim et al., 2014). LPE (18:0) can increase Ca^2+^ concentration in nerve cells through the G2A receptor pathway, resulting in calcium overload (Lee et al., 2015). While, in myocardial infarction induced by a high isoprenaline dose in rat, the enhancement of oxidative stress was observed to be related to the decrease of LPCs [(18:0) and (20:3)] levels (Liu et al., 2013). Increased PC(38:4) levels were found in good responders treated with olanzapine and risperidone. All these above-mentioned studies support our findings that different classes of LPC and LPE were associated with the outcome of treatment in schizophrenia.

This study has a few limitations. First, the sample size is relatively small, which reduces the statistical power of the present study. Second, further study recruiting healthy controls is warranted to verify the clinical value of these predictive biomarkers for the therapeutic efficacy of olanzapine. Third, considering that some patients have been treated with antipsychotics for a short time (less than 14 days) in our study, we cannot completely rule out the possible impact of previous antipsychotics on the results. Fourth, the metabolites were analyzed in plasma rather than in CNS. Future study is warranted to compare the LPC and LPE levels between peripheral (plasma) and CNS (cerebrospinal fluid). Fifth, in a previous study, we published the same cohort of patients and same metabolomic methods (Liu et al., 2021). Although part of methods is similar, the two studies have focused on different clinical problems. Liu et al. focused on the severe side effect of weight gain after treatment with olanzapine. This study focused on the therapeutic response to olanzapine.

Overall, this study provides new evidence that the changes in LPC(18:3) and LPC(20:2) levels were associated with the improvement of positive symptoms, suggesting that LPC may be a potential therapeutic target for olanzapine. Moreover, the baseline levels of LPE(20:5), LPE(18:3) and LPE(22:5) might be highly useful as novel plasma biomarkers for the prediction of therapeutic response in the early stage of schizophrenia. However, this study is limited by the lack of the healthy controls and a small ample size. Replication is warranted in further longitudinal studies with a large sample size to introduce LPC and LPE biomarkers into clinical practice.

## Data Availability

The raw data supporting the conclusions of this article will be made available by the authors, without undue reservation.
